# Continuous Bilateral Serratus Anterior Plane Block: An Effective Analgesic Strategy for Managing Extensive Bilateral Rib Fractures

**DOI:** 10.7759/cureus.77332

**Published:** 2025-01-12

**Authors:** João V Pais, Mariana S Barros, Sónia M Cavalete, Helder P Cardoso

**Affiliations:** 1 Anesthesiology, Unidade Local de Saúde Tâmega e Sousa, Penafiel, PRT

**Keywords:** bilateral rib fractures, continous catheter analgesia, serratus anterior plane block, thoracic analgesia, thoracic trauma

## Abstract

Patients with multiple bilateral rib fractures frequently experience intense pain and limited thoracic mobility, leading to complications such as reduced coughing efficiency, pulmonary infection, and respiratory failure. While thoracic epidural analgesia is often considered the gold standard for pain management in these cases, its use is limited by contraindications and the need for patient positioning that exposes the back. The serratus anterior plane block, guided by ultrasound, offers a promising alternative for managing thoracic pain, particularly for polytrauma patients who cannot sit up or assume lateral positions required for traditional regional blocks. This case report describes a patient admitted to the intensive care unit following a fall that caused multiple bilateral rib fractures. Due to contraindications for epidural analgesia related to coagulopathy, bilateral serratus anterior plane blocks were performed with catheter placement on each side, providing effective pain relief and improving respiratory function.

## Introduction

In polytrauma patients with multiple bilateral rib fractures, severe pain restricts mobility and impairs ventilation, increasing the risk of complications such as pulmonary infections, hypoxia, hypercapnia, and respiratory failure. These challenges can prolong hospital stays, complicate pain management, and lead to increased opioid consumption. Effective pain control is essential, and a multimodal approach combining systemic and regional techniques is recommended to optimize respiratory function and recovery [1]. Thoracic epidural analgesia (TEA) is a commonly used method for managing rib fracture pain [2] but is contraindicated in certain intensive care patients due to coagulopathy and hemodynamic instability. Several regional analgesia techniques are available, including the paravertebral block, erector spinae plane block, and pectoral nerve block [3]. While these techniques have demonstrated efficacy in managing thoracic pain, each has specific limitations. The paravertebral block, for example, shares contraindications with epidural analgesia, is technically complex, carries certain procedural risks, and provides only unilateral analgesia. Additionally, it requires patient mobilization to expose the back, which may not be feasible in polytrauma patients [4]. Similarly, the erector spinae plane block also necessitates patient repositioning to access the back, posing challenges in patients with restricted mobility [5]. The pectoral nerve block may have insufficient dispersion to the needed levels [6]. There is limited literature available on effective alternatives to epidural analgesia for thoracic trauma patients, and this manuscript contributes to this scarce body of evidence by presenting a viable alternative for patients in whom epidural block is contraindicated or impractical.

## Case presentation

A 61-year-old man with complex congenital cyanotic heart disease-including left isomerism, dextrocardia, dextroapex, transposition of the great vessels, atrial and ventricular septal defects, pulmonary infundibular and supravalvular stenosis, and double outlet right ventricle-was admitted to the emergency department following a fall from a ladder. His medical history included type 2 diabetes mellitus and diabetic nephropathy.

On examination, the patient presented with a traumatic brain injury, including an occipital hematoma, right periorbital ecchymosis, and bilateral rib trauma. Imaging revealed fractures of the fourth to seventh ribs on the right and the fifth to seventh ribs on the left, involving the anterolateral thorax. Cranial CT findings were unremarkable. Laboratory results identified acute kidney injury (KDIGO stage 1) and mixed acidemia.

The patient was transferred to the intensive care unit (ICU) for optimized pain management and close monitoring of potential respiratory decline. Initial analgesic management included acetaminophen 1 g every six hours, tramadol 100 mg every eight hours, ketorolac 10 mg every six hours, metamizole 1 g every eight hours, and morphine 2 mg as needed. Despite this regimen, the patient experienced severe, persistent pain that limited chest expansion, reduced mobility, and worsened gas exchange (PaO₂/FiO₂ ratio of 150). High-flow nasal oxygen therapy was initiated at 60 L/min with an FiO₂ of 40%.

The acute pain management team was consulted on the second day of hospitalization. The patient reported a visual analog scale (VAS) pain score of 7/10 at rest and 10/10 during movement, making mobilization impossible. Blood tests revealed thrombocytopenia (platelet count 52,000/mm³), prolonged prothrombin time (PT 14.3 seconds), activated partial thromboplastin time (aPTT 70.4 seconds), and an INR of 1.24, which contraindicated epidural analgesia. Additionally, the erector spinae plane block was considered unsuitable due to the positioning-related discomfort required for back exposure.

**Figure 1:**
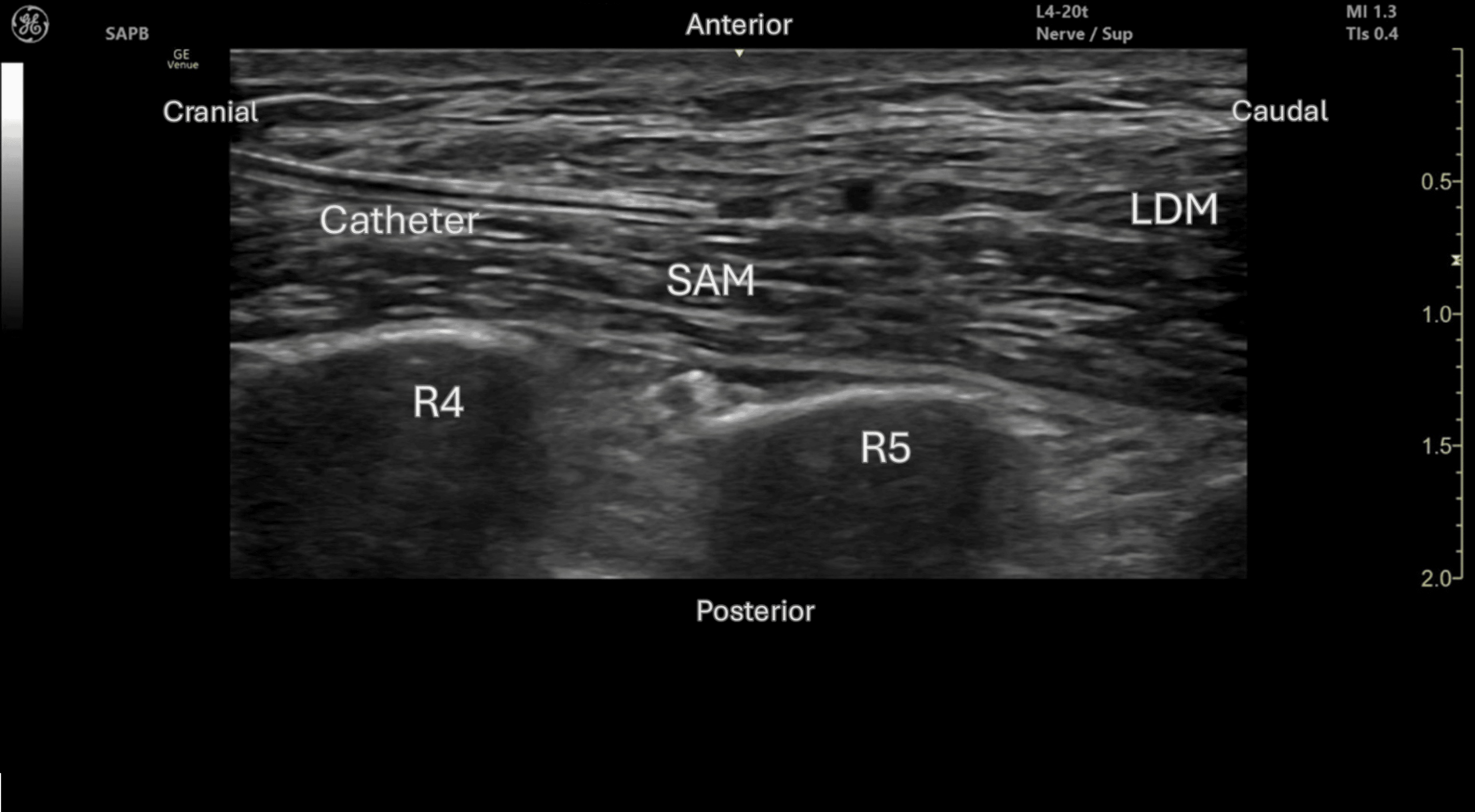
Catheter in serratus anterior plane SAM, serratus anterior muscle; LDM, latissimus dorsi muscle, R4, fourth rib; R5, fifth rib Image provided by the author Mariana S. Barros and edited by Helder P. Cardoso.

After informed consent, a bilateral continuous serratus anterior plane block (SAPB) was performed under aseptic conditions and ultrasound guidance. Using a linear probe (8-13 MHz) and a catheter-over-needle kit (E-Cath® Tsui®, Pajunk, Geisingen, Germany), the fascial plane of the serratus anterior muscle was identified. Following localization, 30 mL of 0.2% ropivacaine was administered bilaterally, and catheters were secured (Figure 1).

Within 20 minutes, the patient’s VAS score decreased to 3/10 during deep breathing, with marked improvement in respiratory mechanics. Bolus doses of 0.2% ropivacaine (15 mL per side) were administered every eight hours. By the next day, ketorolac and morphine were discontinued. The patient was mobilized to a chair, pulmonary rehabilitation was initiated, and oxygen therapy was reduced. On the fifth day, he was transferred to the general ward with only a nasal cannula at 5 L/min and catheters were removed after eight days of well-controlled pain (Table 1).

**Table 1:** Patient respiratory parameters SAPB, serratus anterior plane block; HFNO, high flow nasal oxigenation

Parameters	Normal range	Before SAPB	5 days after SAPB
		HFNO = 60 L/min, FiO_2 _= 40%	Nasal canula = 5L/min
PaO_2_	75 - 100 mmHg	53 mmHg	62 mmHg
PaCO_2_	35 - 45 mmHg	51 mmHg	53 mmHg
Arterial pH	7.35 - 7.45	7.30	7.34
HCO_3_-	22 - 26 mmol/L	25 mmol/L	28.6 mmol/L
Lactate levels	0.5 - 2 mmol/L	0.7 mmol/L	0.6 mmol/L

## Discussion

**Figure 2:**
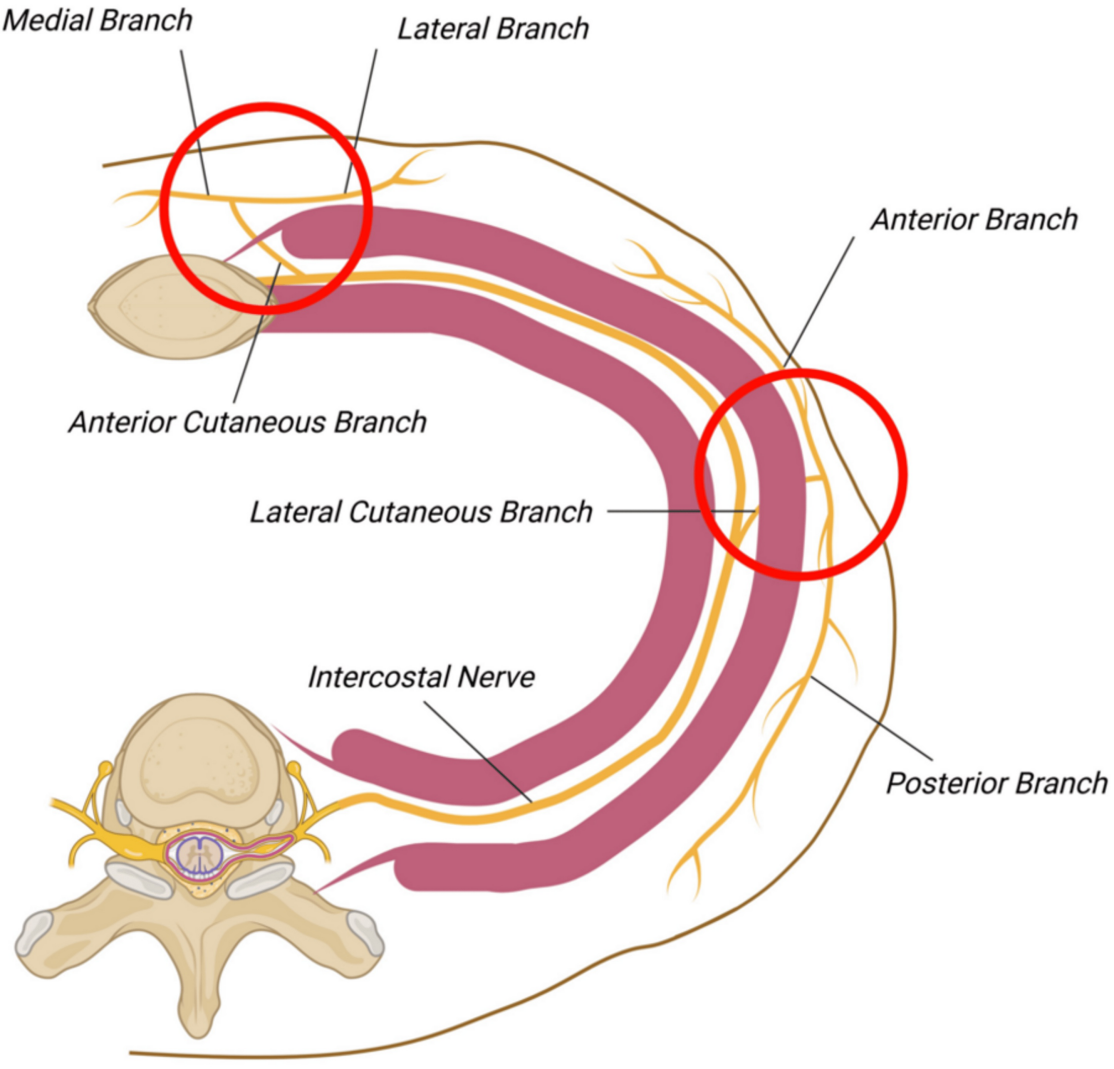
Anatomy of intercostal nerve Image provided by the author João V. Pais.

Effective pain management is critical for patients with multiple rib fractures to reduce pulmonary complications, facilitate recovery, and improve overall outcomes [7]. Achieving sensory blockade of the thoracic wall requires targeting the intercostal nerves, which travel along the inferior margins of the ribs and give rise to lateral cutaneous branches that innervate the chest wall (Figure 2).

The SAPB targets these nerves within the fascial plane between the serratus anterior and latissimus dorsi muscles, providing effective analgesia for the T2-T9 dermatomes, making this block well-suited for chest wall analgesia [8]. While the SAPB is generally regarded as a safe and effective technique, it does carry potential risks and limitations. Local anesthetic toxicity remains a concern, as with other regional anesthesia methods, particularly if systemic absorption occurs. There is also a risk of hematoma formation, especially in patients with coagulopathies or those on anticoagulant therapy. Although rare, pneumothorax is a potential complication due to the proximity of the pleura; however, this risk is significantly reduced when ultrasound guidance is used, as the injection is performed between muscles or above the rib. Additional risks include infection at the injection site and nerve injury, though these are uncommon. Furthermore, SAPB may occasionally result in inconsistent analgesia, necessitating supplementary pain management and potentially delaying crucial interventions to prevent respiratory failure. Despite these limitations, SAPB maintains a favorable safety profile when performed by experienced practitioners in appropriate clinical settings, with literature supporting its reliability, low complication rates, and safety [9-11].

**Figure 3:**
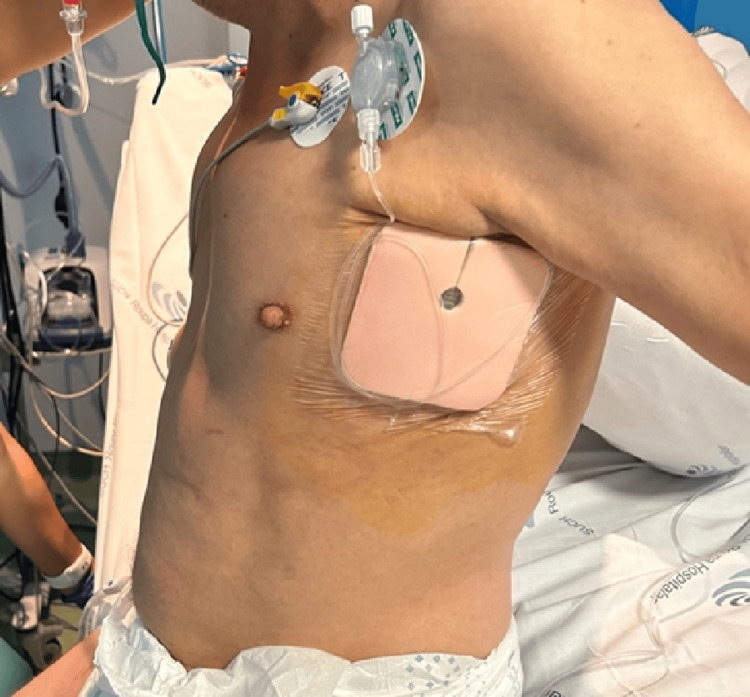
Perineural catheter carefully secured in place. Photo provided by the author Mariana S. Barros with patient consent.

In our case, the placement of two catheters-one on each side-provided prolonged analgesia, ensuring effective pain relief for several days. The minimal patient repositioning required for SAPB made it an excellent option for our polytrauma patient, who could not be easily mobilized to expose his back. SAPB not only provided significant pain relief but also improved respiratory function and reduced dependency on opioids and non-steroidal anti-inflammatory drugs, ultimately enabling the patient to mobilize and recover effectively (Figure 3).

## Conclusions

Ultrasound-guided bilateral continuous SAPB proved to be an effective analgesic approach for managing anterolateral bilateral rib fractures in a patient unable to expose their back. It provided substantial pain relief, enhanced respiratory function, reduced reliance on opioids and non-steroidal anti-inflammatory drugs, and facilitated recovery. This case further supports the growing body of evidence establishing SAPB as a viable and safe alternative to traditional techniques, such as epidural analgesia.
